# Mapping Vaccination Mindsets among UK Residents of Black Ethnicities with HIV: Lessons from COVID-19

**DOI:** 10.1007/s10461-025-04622-0

**Published:** 2025-03-10

**Authors:** Zoe Moon, Lucy Campbell, Zoe Ottaway, Julie Fox, Fiona Burns, Lisa Hamzah, Andrew Ustianowski, Amanda Clarke, Sarah Schoeman, Deirdre Sally, Shema Tariq, Frank A. Post, Rob Horne

**Affiliations:** 1 University College London, London, England; 2 King’s College Hospital NHS Foundation Trust, London, England; 3 King’s College London, London, England; 4 Guys and St Thomas’s NHS Foundation Trust, London, England; 5 Royal Free London NHS Foundation Trust, London, England; 6 Institute for Global Health, University College London, London, England; 7 St Georges University Hospital NHS Foundation Trust, London, England; 8 Manchester University NHS Foundation Trust, Manchester, England; 9 University Hospitals Sussex NHS Foundation Trust, Brighton, England; 10 Leeds Teaching Hospitals NHS Trust, Leeds, United Kingdom; 11 Central and North West London NHS Foundation Trust, London, England; 12 Centre for Behavioural Medicine, School of Pharmacy, University College London, London, England; 13 Brighton and Sussex Medical School, Brighton, United Kingdom

**Keywords:** SARS-CoV-2, COVID-19, Vaccine hesitancy, Beliefs, Attitudes, Black ethnicities

## Abstract

Vaccine hesitancy is a leading threat to public health, but little is known about the beliefs and mindsets that drive vaccine hesitancy, especially among people of Black ethnicities. This study aimed to understand vaccine related beliefs and their relationship with SARS-CoV-2 vaccine uptake in UK residents of Black ethnicities living with HIV. Adults of self-reported Black ethnicities with HIV were recruited at 12 clinics in England. Participants completed questionnaires in clinic, including an adapted version of the Beliefs about Medicines Questionnaire (BMQ) to assess Necessity and Concerns beliefs about the SARS-CoV-2 vaccine. SARS-CoV-2 vaccination status was ascertained through self-report and shared care records. A total of 863 participants were enrolled between June 2021 and October 2022, most of whom (92%) had received at least one dose of the SARS CoV-2 vaccine. After adjusting for age and region of birth, higher perceived need for the vaccine (OR = 2.39, 95% CI = 1.51–3.81), fewer concerns about the vaccine (OR = 0.16, 95% CI = 0.08–0.30), and weaker endorsement of COVID-19 Conspiracy Beliefs (OR = 0.31, 95% CI = 0.19–0.50) were associated with vaccination uptake. Being born outside sub-Saharan Africa was associated with reduced odds of being vaccinated. This study shows the importance of specific beliefs driving vaccine hesitancy and uptake. Further studies should explore the role of these beliefs and mindsets in influencing uptake of other vaccinations, and to work with key stakeholders to explore how to address vaccine hesitancy and improve vaccine uptake in these and other populations.

**Supplementary Information:**

The online version contains supplementary material available at 10.1007/s10461-025-04622-0.

## Background

In 2019, the World Health Organization (WHO) identified vaccine hesitancy as a top ten threat to global health [1]. Vaccine hesitancy has been defined as a delay in acceptance or refusal of vaccines despite availability of vaccine services [1], and as a set of attitudes and beliefs associated with vaccine decision making [2]. Issues around vaccine hesitancy and vaccine confidence are not new, despite the availability of safe, effective vaccines, and the reasons for this are complex and multifactorial [3]. This was bought to the world’s attention during the COVID-19 pandemic, and continues to be an ongoing issue, with growing levels of vaccine hesitancy worldwide [4, 5].

To address vaccine hesitancy, and increase uptake of future vaccine rollouts, we need a better understanding of the beliefs and mindsets that drive hesitancy [6]. We conceptualise mindsets as frames of mind that help people to interpret and make sense of complex information, guiding people’s attention and decision making. Understanding these specific beliefs and mindsets, alongside other factors such as health literacy, allows for development of more effective and tailored health campaigns [7, 8]. Failing to account for these means that health messages are less likely to engage target audiences, less likely to result in behaviour change, and may even have the opposite effect [8, 9]. A recent online study in the US examined COVID-19 vaccine mindsets and showed that intentions to be vaccinated and vaccine uptake were strongly influenced by beliefs related to the personal necessity for and concerns about the SARS-CoV-2 vaccine, which in turn were related to mindsets about vaccines more broadly [10]. This is in line with the Necessity Concerns Framework, a well validated and widely applied framework explaining the key beliefs influencing decisions regarding taking prescribed medications [11, 12, 13]. Little research has applied this framework to decisions about vaccinations [14, 15], especially in racially minoritised groups.

The COVID-19 pandemic has exposed and heightened existing health inequalities. People from racially minoritised groups were at greater risk of COVID-19 infection, morbidity and mortality [16, 17, 18], with a UK Government report showing that the risk of death associated with COVID-19 was up to four times as high for Black men compared to men from other ethnic groups [19]. Alongside this, it was suggested that rates of SARS-CoV-2 vaccine hesitancy and declining the vaccine were higher in people from racially minoritised groups [6, 20, 21], particularly people from Black African and Black Caribbean communities [22, 23, 24]. This is likely driven by both inequity in access to the vaccine (e.g., related to disproportionate bureaucratic processes and reduced flexibility in working hours) [25] and low confidence in vaccines. Research suggests this low confidence in the vaccine may be linked to various factors including personal or collective historic and ongoing poor treatment, structural racism, mistrust of government and health systems, concerns about safety and side effects, and scepticism about effectiveness [3, 6, 26, 27]. A recent review has estimated the prevalence of SARS-CoV-2 vaccine hesitancy to be 34% in people living with HIV, with a higher prevalence among Black people living with HIV [28]. This higher rate was attributed to stigmatisation, and to the social, financial and healthcare related disparities seen during the COVID-19 pandemic [28].

As lack of vaccine uptake has the potential to further exacerbate existing health inequalities, it is essential to gain better understanding of the reasons underlying vaccine hesitancy. These insights can then be used to inform development of tailored interventions to improve vaccine literacy and uptake. The COVID-AFRICA study collected data on SARS-CoV-2 vaccination behaviour and mindsets in people with HIV of Black ethnicities who are living in the UK. These data provide insight into how vaccination uptake may be influenced by an individual’s beliefs about vaccines and the disease that is being vaccinated against.

### Aims

We aimed to understand the views of UK residents of Black African or Black Caribbean ethnicity on COVID-19 and the SARS-CoV-2 vaccine. We sought to better understand beliefs about vaccines, and what might be driving these beliefs, in a sample of people with Black African or Black Caribbean ethnicity living with HIV.

Our specific research questions are:

- What are the beliefs held about SARS-CoV-2 vaccines (Necessity beliefs, and Concerns)?
- Are these beliefs associated with vaccine uptake?
- What is the relationship between COVID-19 Misconceptions and Conspiracy beliefs and vaccine beliefs (Necessity beliefs, and Concerns)?

## Methods

The COVID-AFRICA study was open to all people with HIV who had previously participated in the Genetic Determinants of Kidney Disease in People of African Ancestry with HIV (GEN-AFRICA) study (NCT05685810). The GEN-AFRICA cohort was established between May 2018 and January 2020 and was open to adults of self-reported Black African, Black Caribbean or other Black ethnicities receiving HIV care across 14 sites in England who were able and willing to provide informed consent, demographic and clinical data, and a blood and urine sample for research [29]. GEN-AFRICA participants were approached while attending for HIV care at participating sites (Online Resource 1); they were provided with verbal and written information about the COVID-AFRICA study and if agreeable asked to provide written informed consent. Participants completed paper questionnaires in clinic on aspects of HIV care, COVID-19 illnesses, SARS-CoV-2 vaccination status, beliefs and concerns regarding SARS-CoV-2 vaccines, COVID-19 conspiracy beliefs and misconceptions, and racial inequality beliefs. We obtained routinely collected clinical and laboratory data including HIV viral load and current antiretroviral therapy from the medical records. SARS-CoV-2 vaccination status was ascertained through self-report and review of National Health System shared care records [30]. The study was approved by a National Health Service Research Ethics Committee (21/ES/0047) and the Health Research Authority (IRAS 294887).

### Exposure Variables

Vaccination beliefs were assessed using an adaption of the validated ‘Beliefs about Medicines Questionnaire (BMQ) – Vax (© Prof R Horne; Online Resource 2) [31]. This comprises two scales: (1) Vaccination (Vax) Necessity − 6 items measuring the perceived need for the COVID-19 vaccine (e.g. *A COVID-19 Vaccine will protect me)*; and (2) Vaccination (Vax) Concerns − 10 items assessing concerns about the COVID-19 vaccine (e.g., *I am concerned that a COVID-19 vaccine can give me COVID-19).* These are answered using a five-point Likert scale (strongly agree, agree, uncertain, disagree, strongly disagree). Higher Necessity scores and lower Concerns scores identify more positive beliefs about a COVID-19 vaccine.

To capture COVID-19 conspiracy beliefs, misconceptions, and racial inequality beliefs, we used the Covid Misconceptions and Conspiracy Beliefs questionnaire (CMCQ, © Prof Rob Horne) (Online Resource 3). It includes sixteen questions assessing agreement with common COVID-19 conspiracy beliefs or misconceptions. Three clusters have been identified within the scale (Online Resource 4): (1) Conspiracy beliefs (e.g., *The coronavirus pandemic is not as bad as the government makes it out to be*); (2) Misconceptions (e.g. *COVID-19 only affects older people and is not a problem for younger people*); and (3) Racial Inequality beliefs (e.g., *When it comes to COVID-19*,* black people do not receive the same quality of healthcare as other groups*). Each item is answered using a five-point Likert scale as for the BMQ, with a higher score indicating a greater degree of agreement.

### Outcome Variable

SARS-CoV-2 vaccination status, defined as having had at least one vaccine at the time of enrolment.

### Statistical Analysis

Characteristics of the study population, stratified by SARS-CoV-2 vaccination status, were described and compared using Student’s t-test, analysis of variance (ANOVA), Kruskal-Wallis, or Pearson’s χ^2^ test as appropriate. Mean Vax Necessity and Vax Concerns scores were compared by t-test. Stepwise logistic regression was used to describe the relationship between Vax Necessity/Vax Concerns beliefs, COVID-19 Conspiracy Beliefs and Misconceptions, and vaccination uptake. Demographic variables which were associated (*p* < 0.05) in univariable analysis were included in the multivariate model.

Pearson’s correlation coefficient was used to evaluate the relationship between COVID-19 conspiracy, misconceptions, and racial inequality beliefs, worry about COVID-19 and perceptions around the dangerousness of COVID-19, and Vax Necessity beliefs and Concerns. All analyses were performed using SPSS Version 27.

## Results

Between June 2021 and October 2022, we enrolled 863 participants, 791 (92%) of whom reported receiving at least one dose of an approved SARS-CoV-2 vaccine. The median (IQR) age of participant was 53 (46, 60) years, 54% were female, and 85% were born in Africa or the Caribbean (Table 1). Most had longstanding and well controlled HIV. Participants who were vaccinated were older than those who remained unvaccinated (median age 53.5 [IQR 46.3, 59.7] vs. 48.7 [IQR 42.9, 55.6], X^2^ (1) = 9.724, *p* < 0.001); the rates of vaccination were 97%, 93% and 90% in participants who were born in South/Central, East and West Africa, respectively, 88% in those born in the Caribbean, and 86% in those born in the UK or other countries (X^2^(1) = 13.85 *p* < 0.01). Participants who were vaccinated were also significantly more likely to have known a person who died of COVID-19 (46% vs. 29%, X^2^(1) = 6.58, *p* < 0.01), to think that COVID-19 was dangerous (68% vs. 38%, X^2^(1) = 22.94, *p* < 0.001), and to be worried about COVID-19 (37% vs. 15%, X^2^(1) = 12.01, *p* < 0.001). There were no differences in vaccination rate by gender, time since HIV diagnosis, comorbidity or HIV viraemia status.

**Table 1:** Participant characteristics, stratified by COVID-19 vaccine uptake

		All *n* = 863	Vaccinated *n* = 791	Not Vaccinated *n* = 72	Comparison of vaccinated and not vaccinated
Age, years	Median (IQR)	53.0 (46.2, 59.6)	53.5 (46.3, 59.7)	48.7 (42.9, 55.6)	X^2^ (1) = 9.72**
Sex, female	N (%)	465 (54%)	425 (54%)	40 (56%)	X^2^ (1) = 0.11
Region of birth	N (%)				X^2^(4) = 13.85**
West Africa		283 (33%)	256 (33%)	27 (38%)	
East Africa		181 (21%)	168 (21%)	13 (18%)	
South/Central Africa		208 (24%)	201 (26%)	7 (10%)	
Caribbean	N (%)	57 (7%)	50 (6%)	7 (10%)	
UK/other	N (%)	130 (15%)	112 (14%)	18 (25%)	
Time since HIV diagnosis, years	Median (IQR)	14 (9, 18)	14 (9, 18)	13 (9, 18)	X^2^ (1) = 0.02
Recent CD4 cell count, cells/mm^3^	Median (IQR)	569 (417, 740)	566 (416, 735)	623 (418, 781)	X^2^(1) = 0.92
HIV RNA < 200 copies/mL	N (%)	798 (96)	734 (96)	64 (93)	X^2^(1) = 1.16
BMI, kg/m^2^	Mean (SD)	30.5 (7.3)	30.5 (7.4)	30.2 (7.1)	T(840)=-0.362,
Hypertension	N (%)	290 (34%)	276 (35%)	14 (20%)	X^2^(1) = 7.00**
Diabetes	N (%)	87 (10%)	82 (11%)	5 (7%)	X^2^(1) = 0.87
Cardiovascular disease	N (%)	28 (3%)	19 (4%)	3 (6%)	X^2^(1) = 0.05
COVID experience and perceptions^a^					
Knew a person who died of COVID	N (%)	307 (45)	290 (46%)	17 (29%)	X^2^(1) = 6.58**
Thinks COVID is quite or very dangerous	N (%)	463 (65)	438 (68%)	25 (38%)	X^2^(1) = 22.94***
Is quite or very worried about COVID	N (%)	249 (35)	239 (37%)	10 (15%)	X^2^(1) = 12.01***

^a^ Responses were available for 687 (died), 713 (dangerous) and 708 (worried) participants

UK = United Kingdom; BMI = body mass index; IQR = Interquartile Range. *p < 0.05, ** *p* < 0.01, ***p < 0.001

Vaccinated participants reported higher mean Vax Necessity scores indicating higher perceived need for SARS-CoV-2 vaccination (3.9 [0.8] vs. 2.9 [0.9], t(710)=-10.28 *p* < 0.001), and lower mean Vax Concerns scores indicating fewer concerns about the vaccination (2.3 [0.6] vs. 3.3 [0.7], t(673) = 10.64, *p* < 0.001) than those who remained unvaccinated. They also agreed less strongly with COVID-19 Conspiracy theories (t(704) = 7.14, *p <* 0.001) and COVID-19 Misconceptions (t(707) = 4.83, *p* < 0.001) and were less likely to believe there was a racial bias in healthcare for COVID-19 (t(733) = 2.37, *p* = 0.018) (Table 2).

**Table 2:** Vaccine, COVID-19, and racial inequality beliefs

	All *n* = 863	Vaccinated *n* = 791	Not Vaccinated *n* = 72	t-test
Vax Necessity Score	3.8 (0.8)	3.9 (0.8)	2.9 (0.9)	t(710)=-10.28***
Vax Concerns Score	2.4 (0.7)	2.3 (0.6)	3.3 (0.7)	t(673) = 10.64***
COVID-19 Conspiracy beliefs	2.3 (0.8)	2.2 (0.7)	2.9 (0.9)	t(704) = 7.14***
COVID-19 Misconceptions beliefs	2.1 (0.7)	2.0 (0.7)	2.6 (0.8)	t(707) = 4.83***
Racial Inequality beliefs	2.4 (1.1)	2.3 (1.0)	2.7 (1.1)	t(733) = 2.37**

Data are expressed as mean (SD). *p < 0.05, ** *p* < 0.01, ***p < 0.001

**Fig. 1:**
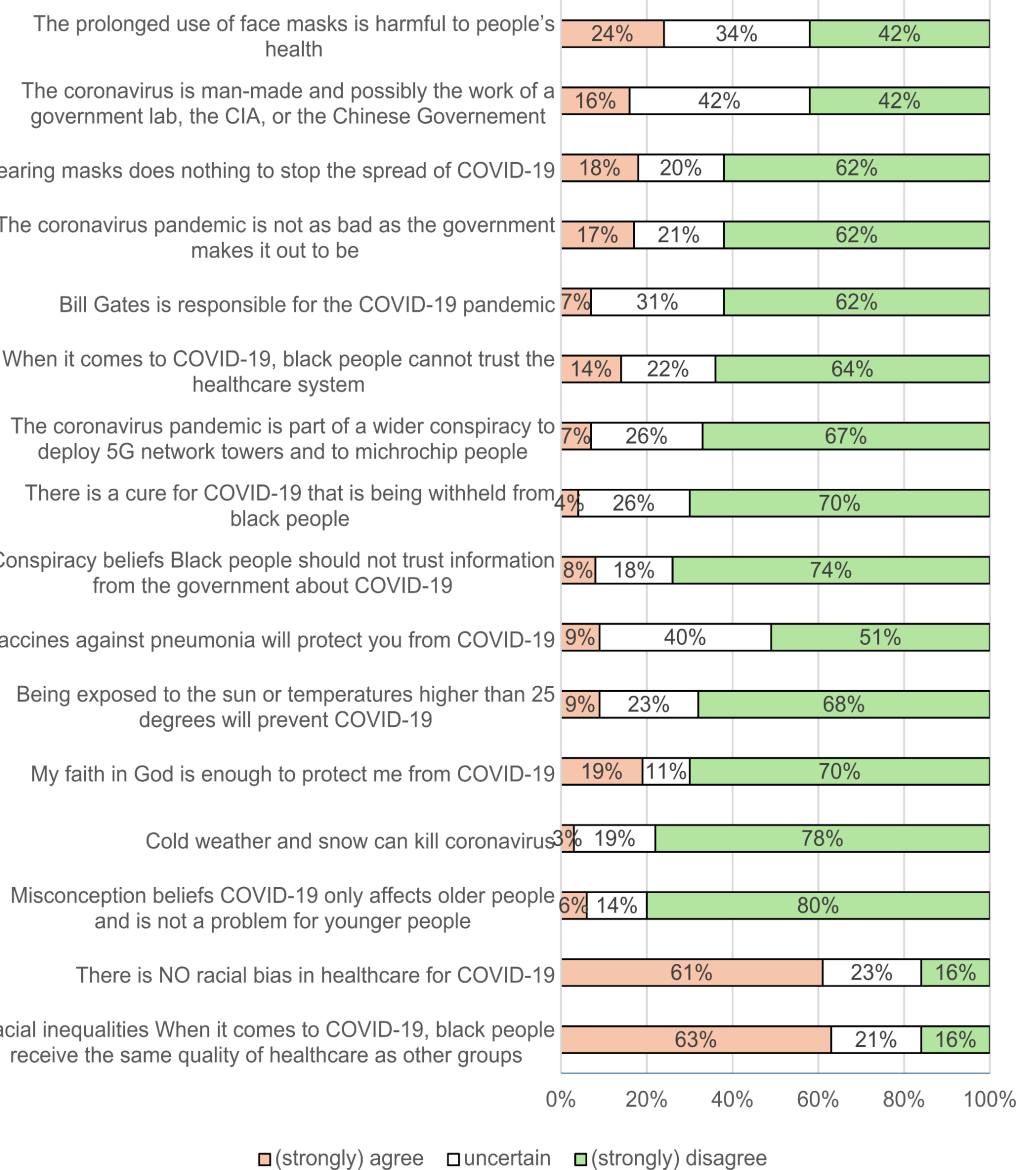
Proportion of participants agreeing, expressing uncertainty, or disagreeing with COVID-19 conspiracy, misconception and racial inequality statements

The responses to the questions in the COVID-19 Misconceptions and Conspiracy Beliefs questionnaire (CMCQ) are shown in Fig. 1. Agreement with COVID-19 Conspiracy Beliefs ranged from 4% (*there is a cure for COVID-19 that is being withheld from Black people*) to 24% (*the prolonged use of face masks is harmful to people’s health*). There were high levels of uncertainty for some items, with 42% being unsure whether ‘*the coronavirus is man-made and possibly the work of a government lab*,* the CIA or the Chinese government’.* Few participants reported misconception beliefs except for faith in God providing protection from COVID-19 (19%). Across two items, most participants (61–63%) did not agree with a racial bias in healthcare for COVID-19, 21–23% expressed uncertainty, and 16% endorsed the presence of a racial bias.

Vax Necessity and Vax Concerns scores were strongly associated with vaccine uptake (Table 3). After adjustment for age and region of birth, a one-point higher Vax Necessity score was associated with greater odds (OR 2.39 [95% CI 1.51, 3.81]) of vaccination uptake while a one-point higher Vax Concerns score was associated with reduced odds (OR 0.16 [0.08, 0.30]) of vaccination uptake. Being born outside sub-Saharan Africa was associated with reduced odds of being vaccinated. COVID-19 Conspiracy beliefs, misconceptions and racial inequality beliefs were also associated with vaccine uptake. Of these, Conspiracy Beliefs remained significantly associated with vaccine uptake (OR 0.31 [0.19, 0.50]) in the adjusted model while COVID-19 Misconceptions and Racial Inequality beliefs were no longer associated with vaccine uptake.

**Table 3:** Factors associated with vaccination uptake

	Unadjusted OR (95% CI)	*p* value		Adjusted OR (95% CI)	*p* value	Adjusted OR (95% CI)	*p* value	
Vax Necessity	4.20 (2.99, 5.90)	< 0.001	2.39 (1.51–3.81)		< 0.001	-		
Vax Concerns	0.11 (0.07, 0.19)	< 0.001	0.16 (0.08–0.30)		< 0.001	-		
COVID-19 Conspiracy beliefs	0.32 (0.22–0.45)	< 0.001	-			0.31 (0.19, 0.50)	< 0.001	
COVID-19 Misconceptions beliefs	0.46 (0.33–0.64)	< 0.001	-			0.99 (0.62, 1.60)	0.998	
Racial Inequality beliefs	0.76 (0.60–0.96)	0.019	-			0.83 (0.62, 1.10)	0.197	
Age								
<40 years	1		1			1		
*≥*40 years	1.95 (1.05, 3.65)	0.04	1.40 (0.54, 3.60)		0.49	1.29 (0.56, 2.96)	0.55	
Country of birth								
South/Central Africa	1		1			1		
West Africa	0.33 (0.14, 0.77)	0.010	0.33 (0.09, 1.12)		0.08	0.49 (0.18, 1.52)	0.154	
East Africa	0.45 (0.18, 1.15)	0.096	0.38 (0.10, 1.39)		0.14	0.52 (0.18, 1.52)	0.234	
Caribbean	0.25 (0.08, 0.74)	0.013	0.17 (0.04, 0.76)		0.02	0.33 (0.09, 1.17)	0.085	
United Kingdom / Other	0.22 (0.09, 0.53)	0.001	0.17 (0.05, 0.60)		0.006	0.24 (0.09, 0.69)	0.008	

Data were analysed using logistic regression

**Fig. 2:**
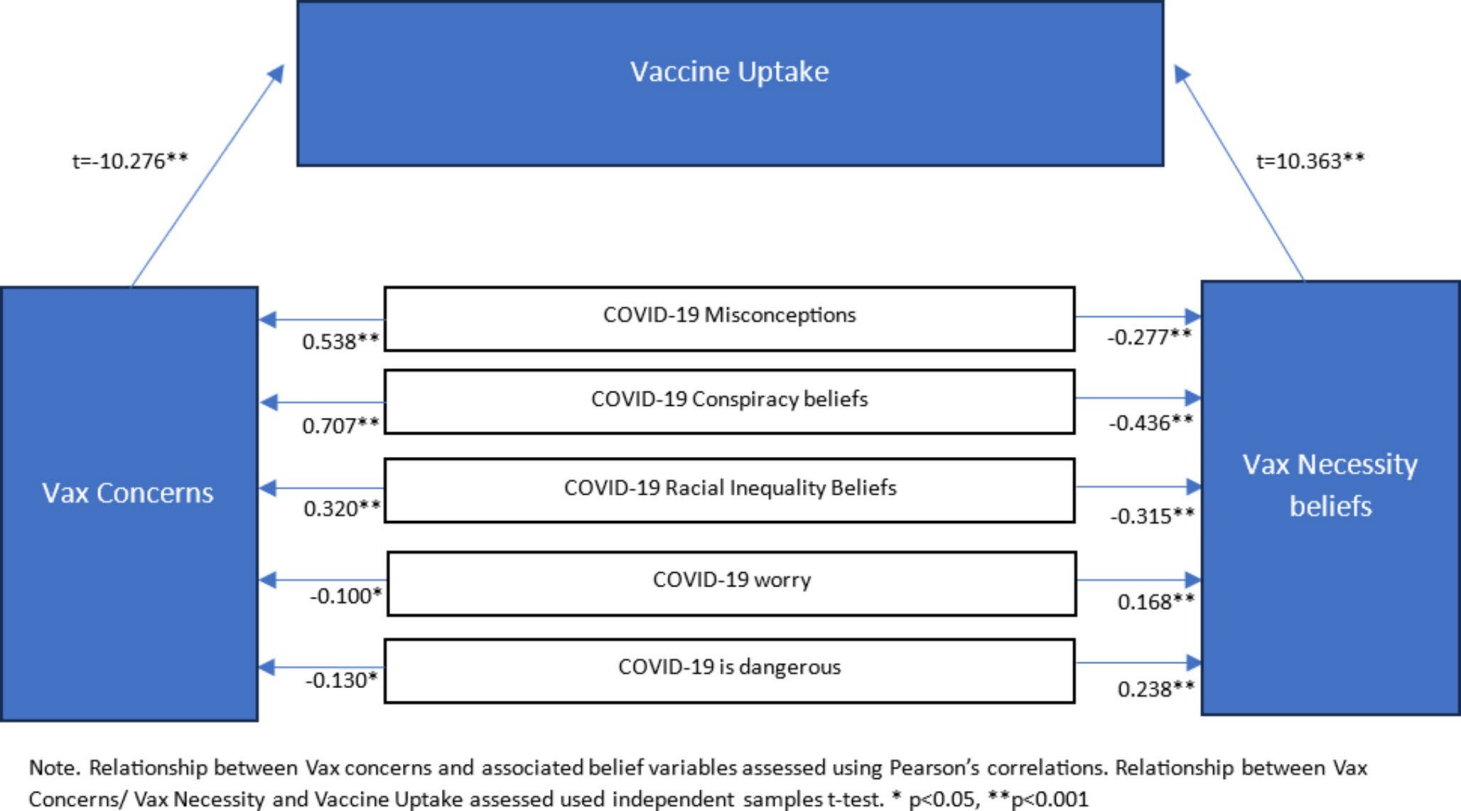
Proposed conceptual model

A proposed conceptual model of the vaccine mindset is presented in Fig. 2. Stronger Vax Necessity beliefs were associated with lower COVID-19 Misconceptions scores (*r*=-0.277, *p* < 0.01), Conspiracy Beliefs (*r*=-0.436, *p* < 0.01), and Racial Inequality Beliefs (*r*=-0.315, *p* < 0.01), as well as higher beliefs that COVID-19 is dangerous (*r* = 0.232, *p* < 0.001) and greater worry about COVID-19 (*r* = 0.168, *p* < 0.001). These stronger Vax Necessity beliefs in turn may have encouraged vaccine uptake (Fig. 2). By contrast, stronger Vax Concerns were associated with higher COVID-19 Misconceptions scores (*r* = 0.538, *p* < 0.01), Conspiracy Beliefs (*r* = 0.707, *p* < 0.01), and Racial Inequality Beliefs (*r* = 0.320, *p* < 0.01), and less worry about COVID-19 (*r*=-0.130, *p* < 0.001). These Vax Concerns may present a barrier to vaccine uptake.

## Discussion

This study provides important insights into beliefs that may contribute to SARS-CoV-2 vaccine hesitancy in a sample of people in the UK living with HIV of Black African or Black Caribbean ancestry. Concerns about the SARS-CoV-2 vaccine and doubts about the perceived need for the vaccine were strongly associated with vaccine uptake, even when controlling for demographic characteristics. Negative beliefs about the vaccine were associated with not thinking COVID-19 was dangerous, not being worried about COVID-19, and endorsing COVID-19 conspiracy beliefs or misconceptions about COVID-19.

Our results showing the importance of vaccine concerns and doubts about the perceived need in SARS-CoV-2 vaccine uptake are consistent with studies in other populations [10], and provide support for the application of the Necessity Concerns Framework in understanding decisions about vaccinations. The relatively high Vax Concerns scores seen in the unvaccinated group is consistent with previous research highlighting a high prevalence of negative attitudes towards SARS-CoV-2 vaccination in the Black community [6, 26, 27]. Previous research has shown that vaccine hesitancy in people from ethnically minoritised groups is partly driven by medical mistrust resulting from previous negative healthcare experiences [21]. This was echoed in the current study, with perceived racial bias in healthcare being associated with concerns about the vaccine. Our results also support previous research showing the negative impact of conspiracy beliefs on vaccine uptake [33]. However, we provide additional insight in showing that these conspiracy beliefs impact and are likely to act through specific beliefs about COVID-19 vaccination (i.e., perceived necessity and concerns). Overall, our results frame vaccine hesitancy not as unreasonable or illogical, but rather as a reflection of common sense decision making by individuals based on their personal feelings, experiences and concerns [34].

The high rate of vaccine uptake among our study participants surpasses those observed in people of Black African or Caribbean ethnicities in the general population in the UK (50–75%) [22, 32] and is only slightly lower than the 88% uptake in the overall COVID-AFRICA study population [30, 41]. This may reflect that our participants with HIV were already well engaged in the UK health system, with generally excellent adherence to antiretroviral therapy. Due to their HIV status and heightened risk of complications and mortality [28], they may have been particularly concerned about COVID-19. Regardless, doubts and concerns about the SARS-CoV-2 vaccine were still strong predictors of vaccine uptake in this population. Lower rates of vaccine uptake and higher rates of negative attitudes may be found in people who are not already well engaged in care. The current study therefore may under-estimate the strength of the association between vaccine beliefs and uptake.

We found the lowest rates of SARS-CoV-2 vaccine uptake among people who were born in the Caribbean, UK or other countries, with the highest rates in those from South/Central Africa. Little research has compared rates across regions within Africa or across the African diaspora. One previous study has shown higher uptake of SARS-CoV-2 vaccines in East and Southern African regions [35]. The lower rates of uptake in people born in the Caribbean should be interpreted with some caution due to the small sample size, but the finding that uptake is lower in Black men and women born in the UK has been reported elsewhere [32] and merits further investigation. The variation in vaccine uptake across people born in the Caribbean and across different regions in Africa demonstrates the importance of not treating Black communities as a homogenous group. These patterns were studied only within a UK sample and other geographic regions that hold a rich African diaspora (e.g., the US) may have different patterns of vaccine uptake. People who were younger were less likely to have received the vaccine, as has been found in other work [36], although the effect of age in our analysis was attenuated and no longer significant in the adjusted model including vaccine beliefs.

Our findings have implications for the development of strategies to reduce vaccine hesitancy in people from ethnically minoritised groups. These insights may be relevant to other vaccination programmes and other populations. Attempts to engage people and change attitudes need to consider the specific beliefs people have about vaccines and how this might affect their decision making. Previous research has suggested that simply debunking people’s opinions is unlikely to be successful [37], and may even backfire and reinforce false beliefs [38]. Instead, trust can be built by maintaining a non-judgemental approach which doesn’t correct opinions or stigmatise people as ‘vaccine hesitant’ or ‘anti-vax’ [34]. Presenting any counter information in a way which acknowledges, respects and resonates with people’s pre-existing beliefs is more likely to be successful [39]. A large UK study has shown that information accessed through the NHS, UK Government or mainstream media which promoted the SARS-CoV-2 vaccine sometimes fuelled people’s suspicions and contributed to further mistrust [40]. Therefore, it is not only the content but the communicating of vaccine messaging which needs to be considered. Providing counter information via trusted networks such as community organisations, religious leaders or other stakeholders can increase receptiveness and spread of the information [21].

There are several limitations to this study. The high rate of vaccine uptake in our study population suggests that individuals who declined vaccination were also more likely to decline participation in the COVID-AFRICA study. Beliefs about COVID-19 and the SARS-CoV-2 vaccines have evolved during the pandemic; as this was a cross-sectional study, we are unable to assess the effects of the COVID-19 mindsets over time. The median age of the population was 53 and the views of younger and older adults were under-represented. The data were cross-sectional, so we are unable to infer the direction of the observed associations. This study looked specifically at the beliefs that influence individual decision making and did not consider the wider social context that these decisions operate in. Nonetheless, the results show the importance of beliefs and mindsets in influencing vaccination uptake. Further studies are now justified to look at the role of these mindsets in influencing uptake of other vaccines and whether these approaches can help understand low vaccine confidence and address vaccine hesitancy ultimately improving vaccine uptake.

## Electronic Supplementary Material

Below is the link to the electronic supplementary material.

Supplementary Material 1

Supplementary Material 2

Supplementary Material 3

Supplementary Material 4

## Data Availability

The database contains personal and sensitive information and is therefore not publicly available. Access to the study data and/or samples is governed by the National Health Service data access policy and those of King’s College Hospital NHS Foundation Trust, the study sponsor. The GEN-AFRICA and COVID-AFRICA studies are open to collaborations, and all requests from researchers who meet the criteria for access to fully anonymized patient level data will be considered. Concepts can be submitted for review to the principal investigator (Prof. Frank Post; email: frank.post@kcl.ac.uk).

## References

[1] Aranda S. Ten threats to global health in 2019. World Health Organisation (WHO). 2019:1–18.

[2] Larson HJ, Gakidou E, Murray CJ. The vaccine-hesitant moment. NEJM. 2022;387(1):58–65. 35767527 10.1056/NEJMra2106441 PMC9258752

[3] Fisher M. Understanding the history of vaccine hesitancy and current mistrust in healthcare. BMJ. 2024; 384. 10.1136/bmj.q539 38453183

[4] Siani A, Tranter A. Is vaccine confidence an unexpected victim of the COVID-19 pandemic? Vaccine. 2022;40(50):7262–9. 36333226 10.1016/j.vaccine.2022.10.061 PMC9618445

[5] Eagan RL, Larson HJ, de Figueiredo A. Recent trends in vaccine coverage and confidence: a cause for concern. Hum Vaccin Immunother. 2023;19(2):2237374. 37526111 10.1080/21645515.2023.2237374 PMC10395254

[6] Hussain B, et al. Overcoming COVID-19 vaccine hesitancy among ethnic minorities: a systematic review of UK studies. Vaccine. 2022;40(25):3413–32. 35534309 10.1016/j.vaccine.2022.04.030 PMC9046074

[7] Abraham C, Kools M. Writing health communication: an evidence-based guide. Sage; 2011.

[8] SAGE. Public health messaging for communities from different cultural backgrounds. https://assets.publishing.service.gov.uk/media/5f5223a7e90e071ec25d3724/s0649-public-health-messaging-bame-communities.pdf Accessed 22 July 2020.

[9] Glass TA, Schoch-Spana M. Bioterrorism and the people: how to vaccinate a city against panic. Clin Infect Dis. 2002;34(2):217–23. 11740711 10.1086/338711

[10] Heller MK, Chapman SCE, Horne R. Beliefs influencing Covid-19 vaccine hesitancy and uptake: an application of the Necessity Concerns Framework. In submission 2024.

[11] Horne R, Weinman J. Patients’ beliefs about prescribed medicines and their role in adherence to treatment in chronic physical illness. J Psychosom Res. 1999;47(6):555–67. 10661603 10.1016/s0022-3999(99)00057-4

[12] Horne R, et al. Understanding patients’ adherence-related beliefs about medicines prescribed for long-term conditions: a meta-analytic review of the necessity-concerns Framework. PLoS ONE. 2013;8(12):e80633. 24312488 10.1371/journal.pone.0080633 PMC3846635

[13] Foot H, et al. The necessity–concerns framework predicts adherence to medication in multiple illness conditions: a meta-analysis. Patient Edu Couns. 2016;99(5):706–17. 10.1016/j.pec.2015.11.004 26613666

[14] Pinho S, et al. Acceptance and Adherence to COVID-19 vaccination—the role of cognitive and emotional representations. Int J Environ Res Public Health. 2022;19(15):9268. 35954625 10.3390/ijerph19159268 PMC9368462

[15] Dale C, et al. The role of medication beliefs in COVID-19 vaccine and Booster Uptake in Healthcare workers: an exploratory study. Healthcare. 2023;11:13. 10.3390/healthcare11131967 PMC10340697 37444801

[16] Apea VJ, et al. Ethnicity and outcomes in patients hospitalised with COVID-19 infection in East London: an observational cohort study. BMJ open. 2021;11(1):e042140. 33455936 10.1136/bmjopen-2020-042140 PMC7813387

[17] Sze S et al. Ethnicity and clinical outcomes in COVID-19: a systematic review and meta-analysis. EClinicalMedicine. 2020;29. 10.1016/j.eclinm.2020.100630 PMC7658622 33200120

[18] Government Equalities Office, Race Dispairty Unit and The Rt Hon Kemi Badenoch MP. Quarterly report on progress to address COVID-19 health inequalities. https://www.gov.uk/government/publications/quarterly-report-on-progress-to-address-covid-19-health-inequalities. Accessed 22 October 2020.

[19] Office for National Statistics. Coronavirus (COVID-19) related deaths by ethnic group, England and Wales: 2 March 2020 to 10. April 2020. 24/05/24] https://www.ons.gov.uk/peoplepopulationandcommunity/birthsdeathsandmarriages/deaths/articles/coronavirusrelateddeathsbyethnicgroupenglandandwales/2march2020to10april2020?hootPostID=b229db5cd884a4f73d5bd4fadcd8959b. Accessed 24th May 2024.

[20] Gibbon S, et al. Uptake of COVID-19 vaccination in a medium secure psychiatric hospital population. BJPsych Open. 2021;7(4):e108. 34059167 10.1192/bjo.2021.924 PMC8167253

[21] Kamal A, Hodson A, Pearce JM. A Rapid systematic review of factors influencing COVID-19 vaccination uptake in Minority ethnic groups in the UK. Vaccines. 2021;9(10). 10.3390/vaccines9101121 PMC8541490 34696228

[22] SAGE. Factors influencing COVID-19 vaccine uptake among minority ethnic groups, 17. December 2020. https://www.gov.uk/government/publications/factors-influencing-covid-19-vaccine-uptake-among-minority-ethnic-groups-17-december-2020

[23] Robertson E, et al. Predictors of COVID-19 vaccine hesitancy in the UK household longitudinal study. Brain Behav Immun. 2021;94:41–50. 33713824 10.1016/j.bbi.2021.03.008 PMC7946541

[24] Perry M, et al. Inequalities in coverage of COVID-19 vaccination: a population register based cross-sectional study in Wales, UK. Vaccine. 2021;39(42):6256–61. 34544601 10.1016/j.vaccine.2021.09.019 PMC8423991

[25] Fuller H, et al. Addressing vaccine hesitancy to reduce racial and ethnic disparities in COVID-19 vaccination uptake across the UK and US. Front Public Health. 2021;9:789753. 34950633 10.3389/fpubh.2021.789753 PMC8688686

[26] Shearn C, Krockow EM. Reasons for COVID-19 vaccine hesitancy in ethnic minority groups: a systematic review and thematic synthesis of initial attitudes in qualitative research. SSM Qual Res Health. 2023;3:100210. 36573229 10.1016/j.ssmqr.2022.100210 PMC9771578

[27] Freeman D, et al. COVID-19 vaccine hesitancy in the UK: the Oxford coronavirus explanations, attitudes, and narratives survey (Oceans) II. Psychol Med. 2022;52:3127–41. 33305716 10.1017/S0033291720005188 PMC7804077

[28] Liu X, et al. COVID-19 vaccine hesitancy among people living with HIV: a systematic review and Meta-analysis. AIDS Behav. 2024;28(7):2183–92. 38625625 10.1007/s10461-024-04344-9

[29] Hung RK et al. The epidemiology of kidney disease in people of African ancestry with HIV in the UK. EClinicalMedicine. 2021;38. 10.1016/j.eclinm.2021.101006 PMC8273351 34286237

[30] Ottaway Z, et al. Clinical epidemiology of COVID-19 in people of black ethnicity living with HIV in the UK. HIV Med. 2024;25(5):614–21. 38213094 10.1111/hiv.13611

[31] Horne R, Weinman J, Hankins M. The beliefs about medicines questionnaire: the development and evaluation of a new method for assessing the cognitive representation of medication. Psychol Health. 1999;14(1):1–24.

[32] Gaughan CH, et al. COVID-19 vaccination uptake amongst ethnic minority communities in England: a linked study exploring the drivers of differential vaccination rates. J Public Health. 2023;45(1):e65–74. 10.1093/pubmed/fdab400 PMC8755382 34994801

[33] Bíró-Nagy A. Szászi Á.J. The roots of COVID-19 vaccine hesitancy: evidence from Hungary. J Behav Med. 2023;46(1):185–200. 35567729 10.1007/s10865-022-00314-5 PMC9106981

[34] Cooper S, et al. Using social media to build confidence in vaccines: lessons from community engagement and social science research in Africa. BMJ. 2024;384:e075564. 38228329 10.1136/bmj-2023-075564 PMC10789190

[35] Njoga EO, et al. Persisting vaccine hesitancy in Africa: the whys, Global Public Health Consequences and ways-Out—COVID-19 Vaccination Acceptance Rates as Case-in-point. Vaccines. 2022;10(11):1934. 36423029 10.3390/vaccines10111934 PMC9697713

[36] Office for National Statistics. Coronavirus and vaccination rates in adults by socio-demographic characteristic and occupation, England: December 2020 to March 2023. 2023.

[37] Chan MS. Albarracín D. A meta-analysis of correction effects in science-relevant misinformation. Nat Hum Behav. 2023;7(9):1514–25. 37322236 10.1038/s41562-023-01623-8 PMC12397989

[38] Peter C, Koch T. When debunking scientific myths fails (and when it does not) the backfire effect in the context of journalistic coverage and immediate judgments as prevention strategy. Sci Comm. 2016;8(1):3–25.

[39] Ruggeri K, et al. Behavioural interventions to reduce vaccine hesitancy driven by misinformation on social media. BMJ. 2024;384:e076542. 38228339 10.1136/bmj-2023-076542 PMC10789192

[40] Woolf K et al. Ethnic differences in SARS-CoV-2 vaccine hesitancy in United Kingdom healthcare workers: results from the UK-REACH prospective nationwide cohort study. Lancet Reg Health Eur. 2021;9. 10.1016/j.lanepe.2021.100180 PMC8287519 34308406

[41] Ottaway Z et al. HIV outcomes during the COVID-19 pandemic in people of black ethnicities living with HIV in England. HIV Med. 2024. 10.1111/hiv.13640 38529684

