# Supplementary material for: Mapping Vaccination Mindsets among UK Residents of Black Ethnicities with HIV: Lessons from COVID-19
Source: AIDS Behav. 2025 Mar 10;29(5):1516–24. doi: 10.1007/s10461-025-04622-0 (PMC12031956; doi:10.1007/s10461-025-04622-0)
Supplement: Supplementary file 1 — Supplementary Material 1 [file 10461_2025_4622_MOESM1_ESM.pdf]

Mapping vaccination mindsets among UK residents of Black ethnicities with HIV: lessons from COVID-19, AIDS & Behaviour, Moon, Z., Campbell L., Ottaway, Z., Fox, J., Burns, F., Hamzah L., Ustianowski, A., Clarke, A., Schoeman, S., Sally, D., Tariq, S., Post, F.A., Horne, R. Corresponding author: Prof Rob Horne, University College London, r.horne@ucl.ac.uk

Online Resource 1. COVID-AFRICA group: Study sites and staff

University Hospitals Sussex NHS Foundation Trust, Brighton (Amanda Clarke [PI], Marion Campbell, Alyson Knott, Lisa Barbour, Vittorio Trevitt)

Chelsea and Westminster NHS Foundation Trust, London (Rachael Jones [PI], Alex Schoolmeesters, Shoreh Ghazali)

Guys and St Thomas's NHS Foundation Trust, London (Julie Fox [PI], Anele Waters, Mariusz Racz, Louise Terry, Kathy Arbis)

King's College Hospital NHS Foundation Trust, London (Frank Post [CI], Zoe Ottaway, Beatriz Santana-Suarez, Leigh McQueen, Lucy Campbell, Bee Barbini, Emily Wandolo, Rachel Hung, Luella Hanbury, Sarah Barber, Chris Taylor, Mary Poulton, Holly Middleditch, Itty Samuel, Candice McDonald, Vladimir Kolodin, Laura Cechin, Larissa Mulka, Amy John, Sally Hawkins, Madelaine Angus, Tim Appleby, Katherine Bainbridge, Hayley Cheetham, Julie Barker, Kate Childs, Rousell Roberts, Nivenjit Kaur, Lisa Shorrock, Kate El Bouzidi, Nisha Patel, Amelia Oliveira, Ayoma Ratnappuli, Kate Flanagan, Daniel Trotman, Nisha Mody, Michael Brady, Killian Quinn, Elizabeth Hamlyn, Gurjinder Sandhu, Verity Sullivan, Naomi Fitzgerald)

Leeds Teaching Hospitals NHS Trust, Leeds (Sarah Schoeman [PI], Gary Lamont, Natasha Calder-Smith, Tadas Mazeika, Kiran Chana)

North Manchester General Hospital, Manchester (Andrew Ustianowski [PI], Gabriella Lindergard, Jan Flaherty, Valerie George, Denise Kadiu, Alice Hendy, Kevin Kuriakose)

Mortimer Market Centre; Central and North West London NHS Foundation Trust, London (Sarah Pett [PI], Mah Jabeen Qamar, Jose Paredes Sosa, Konstantina Tetorou, Michelle Beynon, Adesola Yinka-Ogunleye, Gosala Gopalakrishnan, Irfaan Maan, Claire Mullender)

Newcastle Hospitals NHS Foundation Trust, Newcastle (David Ashley Price [PI], Bijal Patel, Ian McVittie)

Queen Elizabeth Hospital, Woolwich; Lewisham and Greenwich NHS Trust, London (Stephen Kegg [PI], Chloe Saad, Rosa Harrington, Kirsty Cunningham, Mandy Lewis)

Royal Free London NHS Foundation Trust, London (Fiona Burns [PI], Jonathan Edwards, Tom Fernandez, Qayo Egeh, Megan Bailey, Katie Spears)

St George's University Hospital NHS Foundation Trust, London (Lisa Hamzah [PI], Catherine Cosgrove, Hannah Laurence, Ameen Rahman, Katie Toler)

University Hospital Lewisham; Lewisham and Greenwich NHS Trust, London (Melanie Rosenvinge [PI], Claudia Adade, Shelley Campbell)

Africa Advocacy Foundation (Denis Onyango)
