# Supplementary material for: Mapping Vaccination Mindsets among UK Residents of Black Ethnicities with HIV: Lessons from COVID-19
Source: AIDS Behav. 2025 Mar 10;29(5):1516–24. doi: 10.1007/s10461-025-04622-0 (PMC12031956; doi:10.1007/s10461-025-04622-0)
Supplement: Supplementary file 2 — Supplementary Material 2 [file 10461_2025_4622_MOESM2_ESM.pdf]

Mapping vaccination mindsets among UK residents of Black ethnicities with HIV: lessons from COVID-19, AIDS & Behaviour, Moon, Z., Campbell L., Ottaway, Z., Fox, J., Burns, F., Hamzah L., Ustianowski, A., Clarke, A., Schoeman, S., Sally, D., Tariq, S., Post, F.A., Horne, R. Corresponding author: Prof Rob Horne, University College London, r.horne@ucl.ac.uk

Online Resource 3. Questions used to evaluate conspiracy beliefs, misunderstandings/misconceptions, and racial inequality beliefs

	**Conspiracy Beliefs**
**C1**	The coronavirus pandemic is part of a wider conspiracy to deploy 5G network towers and to microchip people
**C2**	The coronavirus pandemic is not as bad as the government makes it out to be
**C3**	Bill Gates is responsible for the COVID-19 pandemic
**C4**	Wearing masks does nothing to stop the spread of COVID-19
**C5**	The coronavirus is man-made and possibly the work of a government lab, the CIA, or the Chinese Government
**C6**	The prolonged use of face masks is harmful to people's health
**C7**	When it comes to COVID-19, black people cannot trust the healthcare system
**C8**	Black people should not trust information from the government about COVID-19
**C9**	There is a cure for COVID-19 that is being withheld from black people
	**Misconceptions**
**M1**	COVID-19 only affects older people and is not a problem for younger people
**M2**	Cold weather and snow can kill coronavirus
**M3**	My faith in God is enough to protect me from COVID-19
**M4**	Being exposed to the sun or temperatures higher than 25 degrees will prevent COVID-19
**M5**	Vaccines against pneumonia will protect you from COVID-19
	**Racial Inequality beliefs**
**R1**	When it comes to COVID-19, Black people receive the same quality of healthcare as other groups*
**R2**	There is NO racial bias in healthcare for COVID-19*

© Copyright Professor Rob Horne

* indicates reverse scoring
