# Supplementary material for: Mapping Vaccination Mindsets among UK Residents of Black Ethnicities with HIV: Lessons from COVID-19
Source: AIDS Behav. 2025 Mar 10;29(5):1516–24. doi: 10.1007/s10461-025-04622-0 (PMC12031956; doi:10.1007/s10461-025-04622-0)
Supplement: Supplementary file 3 — Supplementary Material 3 [file 10461_2025_4622_MOESM3_ESM.pdf]

Mapping vaccination mindsets among UK residents of Black ethnicities with HIV: lessons from COVID-19, AIDS & Behaviour, Moon, Z., Campbell L., Ottaway, Z., Fox, J., Burns, F., Hamzah L., Ustianowski, A., Clarke, A., Schoeman, S., Sally, D., Tariq, S., Post, F.A., Horne, R. Corresponding author: Prof Rob Horne, University College London, r.horne@ucl.ac.uk

Online Resource 4. Factor Analysis of COVID-19 Conspiracy Beliefs and Misconceptions Scale

A principal components analysis using Varimax rotation with Kaiser Normalization indicated a three-factor solution for the COVID-19 Conspiracy Beliefs and Misconceptions Scale: *Conspiracy beliefs* (Cronbach's alpha=0.87), *Medical misunderstanding* (Cronbach's alpha=0.82) and *Racial bias in healthcare* (Cronbach's alpha = 0.81). Kaiser-Meyer-Olkin Measure of Sampling Adequacy was sufficient (0.910) and Barlet's test of sphericity was significant (p<0.001). All factor loadings were above 0.4.

	Component		
	1	2	3
**Component 1: Conspiracy beliefs**			
The coronavirus pandemic is not as bad as the government makes it out to be	.571		
The coronavirus pandemic is part of a wider conspiracy to deploy 5g network towers and to microchip people	.680	.412	
The coronavirus is man-made and possibly the work of a government lab, the CIA, or the Chinese Government	.725		
Bill Gates is responsible for the COVID-19 pandemic	.663	.427	
Wearing masks does nothing to stop the spread of COVID-19	.612		
The prolonged use of face masks is harmful to people's health	.663		
There is a cure for COVID-19 that is being withheld from black people			
When it comes to COVID-19, black people cannot trust the healthcare system	.611		-.328
Black people should not trust information from the government about COVID-19	.652		
**Component 2: Medical misunderstanding**			
COVID-19 only affects older people and is not a problem for younger people		.724	
Vaccines against pneumonia will protect you from COVID-19		.696	
My faith in God is enough to protect me from COVID-19	.378	.585	
Being exposed to the sun or temperatures higher than 25 degrees will prevent COVID-19	.312	.742	
Cold weather and snow can kill coronavirus		.685	
**Component 3: Racial bias in healthcare**			
When it comes to COVID-19, Black people receive the same quality of healthcare as other groups			.896
There is NO racial bias in healthcare for COVID-19			.896
