# Supplementary material for: Mapping Vaccination Mindsets among UK Residents of Black Ethnicities with HIV: Lessons from COVID-19
Source: AIDS Behav. 2025 Mar 10;29(5):1516–24. doi: 10.1007/s10461-025-04622-0 (PMC12031956; doi:10.1007/s10461-025-04622-0)
Supplement: Supplementary file 4 — Supplementary Material 4 [file 10461_2025_4622_MOESM4_ESM.pdf]

Mapping vaccination mindsets among UK residents of Black ethnicities with HIV: lessons from COVID-19, AIDS & Behaviour, Moon, Z., Campbell L., Ottaway, Z., Fox, J., Burns, F., Hamzah L., Ustianowski, A., Clarke, A., Schoeman, S., Sally, D., Tariq, S., Post, F.A., Horne, R.
Corresponding author: Prof Rob Horne, University College London, r.horne@ucl.ac.uk

**Online Resource 2. Questions used to evaluate COVID-19 vaccine necessity and concerns beliefs**

| | |
|---|---|
| | **Questions evaluating vaccination necessity beliefs** |
| **N1** | A COVID-19 vaccine will protect me |
| **N2** | A COVID-19 vaccine will protect me from getting COVID-19 |
| **N3** | I will have a COVID-19 vaccine to get back to normal life |
| **N4** | I will have a COVID-19 vaccine to protect others from COVID-19 |
| **N5** | A COVID-19 vaccine will protect me from getting severe COVID-19 |
| **N6** | I do not need a vaccine because COVID-19 is mild for most people * |
| | |
| | **Questions evaluating vaccination concerns beliefs** |
| **C1** | I am concerned about having a COVID-19 vaccine |
| **C2** | I am worried about the long-term effects of a COVID-19 vaccine |
| **C3** | It is very likely that a COVID-19 vaccine will give me side effects |
| **C4** | I am concerned that a COVID-19 vaccine can give me COVID-19 |
| **C5** | I trust that approved COVID-19 vaccines are safe * |
| **C6** | I am concerned that COVID-19 vaccines contain bits of foetus |
| **C7** | I am concerned that COVID-19 vaccines contain materials from pigs |
| **C8** | COVID-19 vaccines reduce fertility and are a way to control the birth rate in black people |
| **C9** | A COVID-19 vaccine will do more harm than good |
| **C10** | COVID-19 vaccines contain harmful microchips |

© Copyright Professor Rob Horne

* indicates reverse scoring
